# Dental Caries and Other Evils Due to Eating Foods Consisting of Flours, Meals and Grains When the Ferments or Enzymes of the Grain Have Been Impaired, Destroyed or Removed

**Published:** 1912-06-15

**Authors:** Thomas G. Read


### DENTAL CARIES AND OTHER EVILS DUE TO EATING FOODS CONSISTING OF FLOURS, MEALS AND GRAINS WHEN THE FERMENTS OR ENZYMES OF THE GRAIN HAVE BEEN IMPAIRED, DESTROYED OR REMOVED.

BY THOMAS G. READ, L.D.S., Eng., D.M.D., Harv.

In the Royal Dental Hospital Annual Report for 1911, in "A review of the history of dental caries and the present position of the question," Mr. J. Howard Mummery states: "I should scarcely say that the experiments on the different varieties of bread show that there is any bacteriophobe quality in standard bread, they would, I think, simply show that standard bread was not as easily fermented."

The above appears to mean that Mr. Mummery has an idea that all standard breads have similar fermentive qualities. The facts are otherwise; some standard bread very rapidly forms lactic acid during mastication, and some standard bread does not form acid during mastication. What happens depends on whether the diastase of the wheat germ has been destroyed or not. In most cases the diastase is destroyed during milling standard flour; then the bread produced decays the teeth just as badly as ordinary white bread.

He further states:—"As pointed out by Mr. Thomas Read, if bread is properly prepared, the conversion of starch into sugar takes place chiefly during the bread-making, and in the absence of nascent invert sugars in the mouth, there would not be the same amount of fermentation."

Mr. Mummery appears to have a rather confused conception concerning what I have pointed out.

Teeth are not decayed owing to what the baker does, but owing to what the miller does.

When the unimpaired ferments or enzymes of wheat are present in the flour, the diastase of the wheat germ converts starch into glucose during bread-making. When such bread is masticated no acid is formed during chewing, and the glucose passes unchanged into the alimentary canal. Eating such bread therefore does no harm to the teeth.

When the ferments or enzymes of wheat have been impaired or removed during milling the flour, no glucose is formed during bread-making. When such bread is masticated lactic acid is very rapidly formed in the mouth, as the pytalin of the saliva rapidly converts starch into glucose, and from this feshly forming sugar bacteria in the mouth form lactic acid. This acid in its nascent state is not a percentage, but absolute full strength, and has a very powerful decomposive action on tooth tissue.

When such bread is passing through the mouth during mastication, the minute quantities of lactic acid forming are likely to act on the parts of the teeth they are forming close to. Thus each time such bread is eaten the teeth are likely to be decayed a little, without any food resting in the mouth.

Dentists as a body have not paid much attention to the action of breads during mastication.

The meeting of the Odontological Section of the Royal Society of Medicine, on May 22nd last, furnished striking examples of the loose manner in which some dentists handle the subject.

Dr. J. Sim Wallace stated that when stone-milled bread and when white bread are masticated it will be found that both are distinctly alkaline; and Mr. J. F. Colyer stated that he had always thought that Mr. T. G. Read's experiments required confirmation, and he had no doubt that Dr. Sim Wallace's results were correct.

As it is so easy to test bread for acidity before and after mastication, surely Mr. J. F. Colyer and Dr. Stanley Colyer, before writing their book, "Dental Disease in its Relation to General Medicine," should have been aware that all breads are acid; some form acid during mastication, some do not, and bread is never alkaline; they should also have been acquainted with the changes taking place in different varieties of flours during bread-making, and the changes in various breads during mastication. Dentists as a body have not troubled to study elementary facts relating to different varieties of breads that all dental students should be conversant with during their first year at a dental school. Had dentists been more interested in the chemical changes that take place with different varieties of breads, instead of a dental surgeon to the Royal Dental Hospital rising to agree with the statement that breads are alkaline, it is possible the idea would have been greeted with merriment. Dr. Wallace's theories of dental caries appear founded more on thought than on verification. He imagines that food resting around the teeth is a frequent cause of dental caries, in fact the only cause.

Owing to not being aware that some breads form lactic acid so rapidly during mastication that harm is done to the teeth without any food lodging in the mouth, he has magnified a minor risk of caries into a major one. He appears not to have noticed that it is usually after teeth have commenced to decay that food is likely to lodge in the mouth. Food resting around the teeth is more a sign than a cause of dental caries.

My investigations and experiments have convinced me that more teeth are decayed by lactic acid formed from bread during mastication than from all other causes, and if only bread that does not form lactic acid during mastication were eaten, there would be an enormous decrease in the number of teeth lost owing to dental caries.

The change in the method of milling flour that has taken place during the past fifty years is the cause of the great increase in dental caries during recent years. Bread made from flour containing the ferments of wheat contains glucose, and it is a matter of remaining hours in the mouth for it to form lactic acid. Mr. Kenneth Goadby states that by means of mouth bacteria, in the majority of instances, a 2 per cent. solution of glucose or maltose, in peptonic water, having a reaction neutral to litmus, will show an acid reaction in five or six hours.

Bread made from flour not containing the ferments of wheat does not contain glucose, and it is a matter of seconds during mastication for the ptyalin of the saliva to convert starch into glucose, and for bacteria in the mouth to form this forming sugar into lactic acid.

Dr. Leonard Hill in his experiments last year at the London Hospital, using standard bread made from flour when the ferments of the wheat had been destroyed during milling, and white bread made from flour when the ferments had been removed during milling, having chewed each bread for two minutes, found that lactic acid had been developed in very similar quantities, during mastication, by each bread.

If any kind of bread be chewed and then kept in an incubator at the temperature of the mouth for twenty-four hours, on opening the drawer the odour of lactic acid is very perceptible. If a number of sound teeth be obtained and the crowns imbedded in freshly-chewed breads, and placed in an incubator, the teeth being daily removed, washed and replaced in freshly-chewed breads, after some weeks it will be found that opaque white specks appear in the enamel, as if those spots had been bruised. In time the whole surface of enamel will become an opaque white. The disintegrated enamel crumbles away and when the dentine is exposed, it will be found it has been softened, and with an excavator can be easily removed in flakes.

The above experiments are easy, but not very instructive concerning dental caries, as when chewed bread remains in the mouth it is all the time more or less washed with saliva; whereas in an incubator the bread is not disturbed except when replaced by a fresh supply.

If the above experiments be repeated in a warm room, without using an incubator, removing the teeth from the chewed breads, wiping them and replacing them in freshly-chewed breads, about as frequently as the mouth is refilled with food during eating, the nearer the conditions approach to those of the mouth during mastication the more rapidly chewed bread made from flour not containing the ferments of wheat will disintegrate the teeth, and the less effect will chewed bread made from flour containing the unimpaired ferments of wheat have on the teeth. The teeth should not be allowed to remain in stale chewed bread, and at the end of a series of chewings they should be kept in distilled water until commencing another series of chewings.

Since the chief requirements to conduct the above experiments are some sound teeth, supplies of breads and someone with sufficient persistence and patience to frequently chew breads to renew the supplies the teeth are imbedded in, it is possible, for any dentist in the true interest of his patients to satisfy that my statements for many years concerning breads and dental caries are absolutely correct.

The risk of slowly causing the teeth to decay have been enormously increased during recent years owing to most of the breads now supplied to the public forming lactic acid during mastication.

To brush the teeth after eating bread made from flour not containing the ferments of wheat, is on a par with locking a stable door after the horse is stolen. The harm already done is not reduced.

If more attention had been paid years ago to my statements on August 6th, 1901, at the annual general meeting of the British Dental Association held at the Examination Hall of the Royal College of Physicians and Surgeons, many of the hundreds of thousands of our children who now suffer from decayed teeth, might have had sound dentitions.

Some of our leading men of science are endeavoring to discover the reason bread loses nutritive value.

It is most important in the interests of public health that the ferments of wheat should not be impaired, destroyed or removed by millers during milling.

Cases of impaired health in animals and birds due to the ferments in grains and meals having been impaired or destroyed have come under my notice.

In fact, it was owing to some young pigs that had been reared on locally ground barley-meal, being fed on stale imported barley-meal, that first, in the early nineties, directed my investigations to the importance of the ferments of grain for the maintenance of good health. This resulted from having a circular trough with eight divisions and weaning nine pigs. Thus at feeding time one pig was unable to be eating, and waited until one at the trough raised the head, then the outsider captured the place until another took it. It amused me to watch the excitement, until one day when the pigs were fed they showed little interest in the food. There being much swine fever in the neighborhood, I wondered if this was an early symptom of an attack, but the stockman ridiculed such a suggestion, owing to the curly tails, erect ears and sleek coats of the pigs. He suggested the pigs did not like the food; owing to the barley grown on the farm and ground into meal at a local mill having been all used up, a supply had been obtained elsewhere. This he thought was foreign ground meal. The pigs did not do well on their change of meal, and some weeks after their tails were hanging straight down, their ears flopping like those of elephants, and their coats dull. Then one pig died, and a Government inspector came and took away parts of the dead pig, and later reported it was not swine fever. Then the pigs were again fed on barley-meal grown and ground in the neighborhood, and the remaining eight pigs soon recovered, were fatted and sent to market. The dealer who supplied the barley-meal which the pigs did not like, admitted it was imported, but stated it had no musty or objectionable odor. It had very little odor.

When the lid of a bread-pan is lifted it is easy to smell if the ferments were in the flour the loaf was made from. Meals have very similar odors when the ferments are not destroyed. The ferments of the imported barley meal had been destroyed.

The young pigs developed very similar symptoms, while fed on the imported meal, to those developed by the pigeons fed on white bread last year at the Liverpool School of Tropical Medicine, in the experiments conducted by Drs. Edie and Simpson.

Cattle do badly if fed on grain or meal that has had the ferments injured or destroyed.

Some years ago the late Lord Stanley, of Alderley, wrote me concerning some bullocks on a Welsh farm suffering from pellagra. The local veterinary surgeons were baffled as to the cause. A man from South America saw the poor beasts and said, "The pellagra is due to giving the bullocks broken maize that has lost its vitality. Give them sound corn, and they will soon be all right." This was done and they soon recovered.

When residing at Heathfield, Sussex, I was Chairman of the Sussex Poultry Association, an organization to safeguard the interests of chicken fatteners. This brought me into close touch with the leading fatteners of the industry, and placed me in a position to become conversant with much connected with chicken fattening. Since chickens during fattening have no exercise, and should remain only twenty-one days in the crates, it is most important that the food, besides being conducive to fattening, must also tend to keep the birds in the highest possible conditions of health. The diet of fattening chickens is Sussex ground oats, sharp, milk and fat. When the ferments of the oats are injured or destroyed the chickens do not do well. What is injurious to the health of animals and birds must also be injurious to the human race.

The health and the physique of the nation has been greatly injured by millers in some cases destroying the ferments of wheat, and in others removing them when making flour.

In the interests of public health an Act of Parliament should be passed, making it illegal for millers to impair, destroy or remove the ferments of wheat when making flour for bread-making.—*Dental Record.*

